# Comparison of Five 2nd-Generation Supraglottic Airway Devices for Airway Management Performed by Novice Military Operators

**DOI:** 10.1155/2015/201898

**Published:** 2015-10-01

**Authors:** Tomas Henlin, Michal Sotak, Petr Kovaricek, Tomas Tyll, Lukas Balcarek, Pavel Michalek

**Affiliations:** ^1^Department of Anesthesia and Intensive Medicine, 1st Medical Faculty and University Military Hospital, U Vojenske Nemocnice 1, 169 02 Prague, Czech Republic; ^2^Department of Anesthesia and Intensive Medicine, 1st Medical Faculty and General University Hospital, U Nemocnice 2, 128 08 Prague, Czech Republic; ^3^University of East Anglia, Norwich Research Park, Norwich NR4 7TJ, UK

## Abstract

*Objectives*. Five different second-generation supraglottic airway devices, ProSeal LMA, Supreme LMA, i-gel, SLIPA, and Laryngeal Tube Suction-D, were studied. Operators were inexperienced users with a military background, combat lifesavers, nurses, and physicians. *Methods*. This was a prospective, randomized, single-blinded study. Devices were inserted in the operating room in low light conditions after induction of general anesthesia. Primary outcome was successful insertion on the first attempt while secondary aims were insertion time, number of attempts, oropharyngeal seal pressure, ease of insertion, fibre optic position of device, efficacy of ventilation, and intraoperative trauma or regurgitation of gastric contents. *Results*. In total, 505 patients were studied. First-attempt insertion success rate was higher in the Supreme LMA (96%), i-gel (87.9%), and ProSeal LMA (85.9%) groups than in the Laryngeal Tube Suction-D (80.6%) and SLIPA (69.4%) groups. Insertion time was shortest in the Supreme LMA (70.4 ± 32.5 s) and i-gel (74.4 ± 41.1 s) groups (*p* < 0.001). Oropharyngeal seal pressures were higher in the Laryngeal Tube Suction-D and ProSeal LMA groups than in other three devices. *Conclusions*. Most study parameters for the Supreme LMA and i-gel were found to be superior to the other three tested supraglottic airway devices when inserted by novice military operators.

## 1. Introduction

Combat lifesavers in the Czech Army have already been trained for several years in easy and effective airway management during practicing medicine in the field. Generally, the algorithms of the Tactical Combat Casualty Care (TCCC) are applied [1]. The role of supraglottic airway devices (SADs) in these recommendations is not completely clear. As is well known from civilian prehospital medicine, tracheal intubation is a relatively complicated and risky procedure in the hands of nonanesthetic personnel such as paramedics [2, 3]. Similar conditions apply for combat lifesavers (CLS). SADs may provide a more patent airway than the nasopharyngeal airways currently recommended by the TCCC; however, they can be used only if they are tolerated such as in severely injured unconscious victims. In 2012, supraglottic airway devices were recommended for consideration in the TCCC [4]. Following a literature search, there currently is no comparison of currently available SADs which has been published for their use in military medicine. Theoretically, those SADs which can drain gastric contents using a separate channel or those with other aspiration protection mechanisms (compartment for storage of gastric contents), classified as 2nd-generation SADs, are more advantageous in prehospital medicine (with nonfasted patients) than the more simple 1st-generation devices, which do not possess any protective mechanism against aspiration [5]. Therefore we aimed to compare five different SADs with a protection mechanism against aspiration, ProSeal laryngeal mask airway (PLMA) [6], Supreme laryngeal mask airway (SLMA) [7], i-gel [8, 9], Streamlined Liner of the Pharyngeal Airway (SLIPA) [10], and Laryngeal Tube Suction-D (LTS-D) [11], in the settings of prospective randomized trial in simulated low light conditions performed by inexperienced military operators.

## 2. Methods

### 2.1. Study Design

This study was designed as randomized, prospective, and single-blinded (patient side). Ethical approval was obtained from the Local Ethical Committee (IRB), number 80-76/39-2012-UVN. The research was performed in full accordance with the Helsinki Declaration and the study was registered with a public database (R&D IS of the Czech Republic).

### 2.2. Study Setting and Population

All patients scheduled for elective procedures under general anesthesia during the study period and meeting the inclusion criteria were invited to participate in this study. The study setting was University Military Hospital in Prague, Czech Republic. Approximately 10 000 procedures under general anesthesia are performed in this hospital annually. The study period lasted from August 2012 till December 2013.

### 2.3. Study Protocol

**Figure 1:**
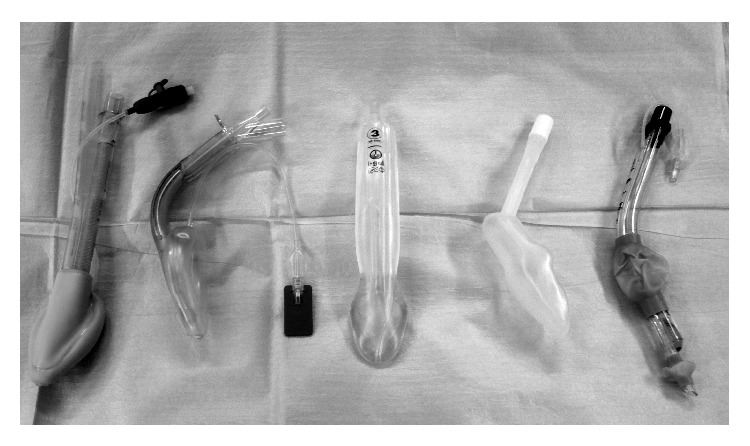
SADs used in the study. From the left: ProSeal LMA (PLMA), Supreme LMA (SLMA), i-gel, SLIPA, and LTS-D.

All patients received the Study Information Pack and had the opportunity to discuss their participation with the researchers in advance. The inclusion criteria were elective surgery with an indication for an SAD insertion, age more than 18 years, and procedures with an expected duration of less than 2 hours. Exclusion criteria were increased risk of aspiration of gastric contents, emergency procedures, operations with an expected duration of more than 2 hours, obesity (BMI > 40 kg/m^2^), history or prediction of difficult laryngoscopy, pregnancy, and edentulous subjects. The randomization process was performed using a randomization list created with the freeware http://www.psychicscience.org/ and patients were randomized after signing the informed consent with the code provided in sealed envelopes. The following SADs were used in patients, ProSeal laryngeal mask airway (Laryngeal Mask Company Ltd., Mahé, Seychelles), Supreme laryngeal mask airway (Laryngeal Mask Company Ltd., Mahé, Seychelles), i-gel (Intersurgical Ltd., Maidenhead, UK), Streamlined Liner of the Pharyngeal Airway (SLIPA) (Curveair Ltd., London, UK), and Laryngeal Tube Suction-D (VBM Medical, Sulz, Germany) (Figure 1), known as King LTS-D device in the United States. The main features of these SADs are highlighted in Table 1.

The primary outcome of the study was to evaluate the first-attempt insertion success rates of the devices and compare these between the groups. Secondary outcomes were evaluated as follows: time needed for successful insertion, number of insertion attempts, oropharyngeal seal pressure (OSP), ease of insertion, fibre optic check of the vocal cords through the devices, and presence of perioperative oropharyngeal trauma or gastric content aspiration/regurgitation. etCO_2_ was controlled at 1, 5, and 10 minutes after insertion as a marker of efficient ventilation.

### 2.4. Anesthesia

**Figure 2:**
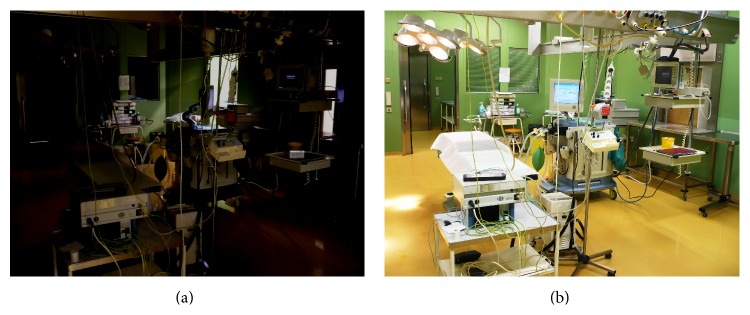
Conditions in the operating room without (a) and with (b) an artificial light.

All patients were premedicated using oral midazolam at a dose of 7.5 mg prior to surgery. Induction of general anesthesia was performed without the use of artificial light in gloomy conditions (Figure 2), using propofol (2–2.5 mg·kg^−1^) and a continuous infusion of remifentanil (1-2 mcg·kg^−1^·min^−1^) until the loss of verbal contact and eyelash reflexes. Anesthesia was maintained using isoflurane in an air : oxygen mixture whereas analgesia was maintained with a continuous infusion of remifentanil at a rate of 0.2–1.0 mcg·kg^−1^·min^−1^. No muscle relaxants were given as part of the study protocol.

### 2.5. Airway Management

SADs were inserted only by inexperienced operators working or undergoing training in a military hospital. They were defined as users who have not inserted an SAD more than five times in real patients. The operators included combat lifesavers, military paramedics, nurses, surgical scrub nurses, or junior doctors at the beginning of their training. All participants were employed by the Czech Army and completed Basic Airway Skills Course according to their job competencies. The course agenda included bag-mask ventilation, insertion of oral/nasal airways, insertion of supraglottic airway devices, tracheal intubation, and surgical cricothyrotomy. Majority of training is performed on manikins or simulators. In regard of SADs all novice operators observed instructive insertion of these devices, had opportunity to insert them on manikins, and were apprized of each SAD prior to its insertion. A consultant anesthesiologist was always present in order to deal with failures or complications. The consultant performed a short presentation about the SAD to the operator prior to insertion, explaining its preparation, lubrication of the device, insertion technique, fixation, and evaluation of its efficacy. Size of the devices was selected according to the weight of patients and manufacturer recommendations. Level of consciousness was evaluated in all patients after induction of general anesthesia using the AVPU score and Glasgow Coma Scale. The devices were inserted only in patients who did not react to painful stimulus, forced jaw thrust. Failure to effectively insert an SAD was defined as impossibility to achieve tidal volumes of 4 mL·kg^−1^ and to eliminate CO_2_ (etCO_2_ over 6.5 kPa) at 10 minutes despite repeated insertion attempts. If the operator was not able to achieve successful ventilation on five insertion attempts, tracheal intubation was performed as a rescue procedure by a supervising consultant. Insertion time was defined as time needed for SAD preparation (removal from the package, lubrication), its insertion, cuff inflation (if applicable), and confirmation of effective ventilation with a visible etCO_2_ tracking on the monitor and this started immediately after finishing initial bag-mask ventilation. The cuffs of inflatable devices (PLMA, SLMA, and LTS-D) were inflated according to the manufacturer recommendations. Correct position of PLMA, SLMA, and i-gel was also confirmed using “suprasternal notch” and “bubble” tests. Patients were artificially ventilated using pressure control ventilation (PCV mode) with a target tidal volume of 7 mL·kg^−1^. Other parameters of ventilation were respiratory rate 12–16 per minute, inspiration/expiration ratio 1 : 2, and PEEP 4 cm H_2_O. End-tidal CO_2_ level was maintained between 4.7 and 5.3 kPa. An airway leak test was performed at 5 min after insertion; pressure limit was set to 40 cm H_2_O, the APL valve was fully closed, and air flow was set to 3 L·min^−1^. Oropharyngeal seal pressure (leak pressure) was defined as the pressure inside the system when the first sounds were audible above the larynx using a stethoscope [12].

### 2.6. Fibre Optic Evaluation

Fibre optic check of the SAD position was performed through the device using a flexible fibrescope. The following scoring system was used: (1) full view of glottis, (2) vocal cords, arytenoids, and inferior surface of epiglottis visible, (3) only superior surface of epiglottis visible, and (4) no part of epiglottis or larynx visible [13].

### 2.7. Ease of Insertion

This was a subjective evaluation performed by the operator, for which a five-point Likert scale was used ((1) very easy insertion, (2) easy insertion, (3) neither easy nor difficult insertion, (4) difficult insertion, and (5) very difficult insertion).

### 2.8. Airway Complications

The devices were carefully examined after removal for any signs of blood or gastric fluid. The oral cavity of patients was evaluated for bleeding or signs of regurgitation after removal of the SAD. Postoperative complaints were not evaluated.

### 2.9. Sample Size and Data Analysis

The sample size was calculated for an alpha-error of 0.05 and a power of 80% (beta-error of 0.2) to detect a 20% difference in insertion success rate on the first attempt. 65% success rate was considered as a baseline according to the study of Ragazzi et al. [14]. It was calculated that a minimum of ninety patients in each group should be enrolled. We have chosen to include at least one hundred patients in each group in order to compensate for patients lost to follow-up. In total 520 allocations were created using randomization freeware. Statistical analysis was performed by an independent consultant statistician. All data were first tested for their normal distribution using the Shapiro-Wilk test of normality. According to the data distribution, nonparametric (Kruskal-Wallis and Mann-Whitney tests) or Fisher's exact tests were employed. SPSS 13.0 (SPSS Inc., Chicago, IL) statistical software was used for data analysis. *p* values less than 0.05 were considered as significant.

## 3. Results

### 3.1. Sample

**Figure 3:**
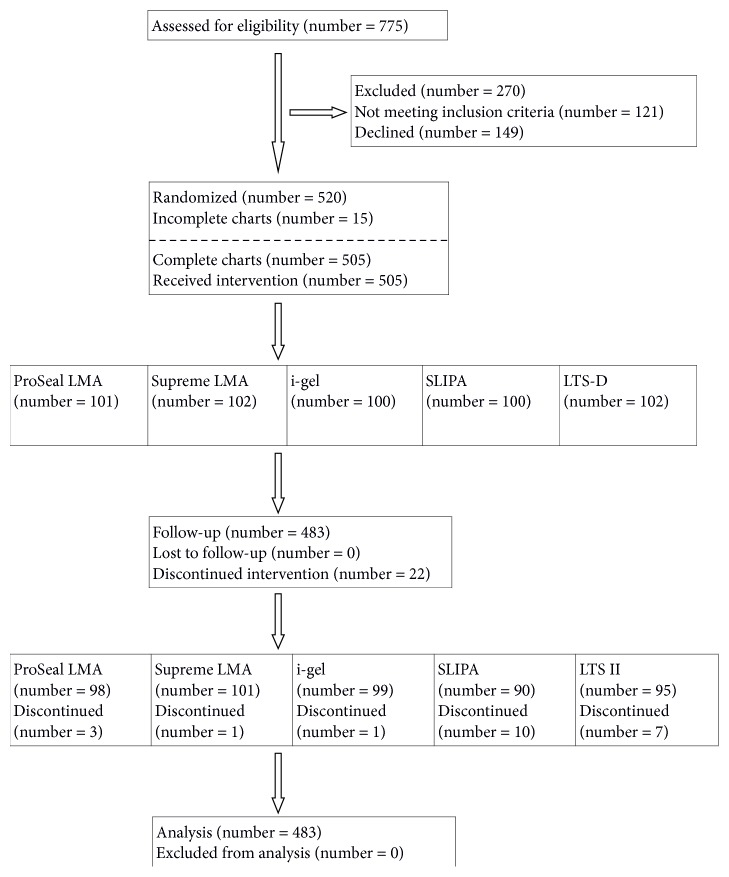
CONSORT 2010 flow diagram of the study.

In total 520 randomization codes were created and finally 505 patients were included in the study. Fifteen patients dropped out during the study period or their charts were incomplete.  Figure 3 demonstrates flow of the study. There were no statistical differences in demographic parameters between the groups.

### 3.2. Primary Outcome

Insertion success rate on the first attempt varied between the devices (Table 2). These success rates were highest in the SLMA group (95.1%), followed by i-gel (87%), PLMA (84.2%), and LTS-D (77.5%). SLIPA demonstrated the lowest first-attempt insertion success rate at 66%. First-attempt insertion success rate of the SLMA was significantly higher than in the PLMA (*p* = 0.012), SLIPA (*p* = 0.0001), or LTS-D (*p* = 0.0004) groups. Similarly, both PLMA and i-gel showed higher insertion success rate on the first attempt than SLIPA device (*p* = 0.003 and *p* = 0.0007, resp.).

### 3.3. Secondary Outcomes

The total insertion success rate was similarly high for the SLMA (99%), PLMA (97%), and i-gel (99%), whereas the LTS-D (93.1%) and SLIPA (90%) showed slightly lower numbers. However, statistically significant difference was achieved only between SLMA and SLIPA (*p* = 0.005), i-gel and SLIPA (*p* = 0.01), and PLMA and SLIPA (*p* = 0.049), respectively.

Time for successful insertion was shortest in the SLMA (70.4 ± 32.5 s) and i-gel (74.4 ± 41.1 s) groups in comparison with the other three devices (Table 3). Insertion of the PLMA, SLIPA, and LTS-D was significantly prolonged (*p* < 0.001) when compared with the SLMA and i-gel. The highest oropharyngeal seal pressures were achieved with the PLMA and LTS-D devices. The remaining three devices exhibited significantly lower seal pressures: *p* < 0.001 (Table 3).

Best view of the glottis, as confirmed by fibre optic evaluation, was achieved with the i-gel airway (88.7% of grade 1 or 2) while this was lowest in the LTS-D group (only 62.2% of grade 1 or 2): *p* < 0.001. Significant differences in the view of the glottis were also found between the i-gel and SLMA (*p* < 0.05) or SLIPA (*p* < 0.05) (Table 4). Fibre optic evaluation was performed in 465 patients (96.3%); the remaining 18 devices were not assessed due to unavailability of the fibrescope. The efficacy of ventilation was evaluated as better in the PLMA, SLMA, and i-gel groups as compared with the remaining two devices (*p* < 0.05).

Regarding ease of insertion, the participants reported that the SLMA was the easiest device to insert, 62% very easy and 31% easy, while the LTS-D and SLIPA were most difficult to introduce, less than 50% of very easy or easy insertions (Table 5).

Perioperative complications such as blood on the device on removal or regurgitation/aspiration of gastric contents occurred only in 18 patients (3.8%). Blood on the device after removal was found in one patient in the PLMA (1%), SLMA (1%), and i-gel (1%) groups, in five patients managed with the LTS-D (4.9%), and in ten patients in the SLIPA group (10%). One patient significantly regurgitated through the gastric lumen of the PLMA but did not aspirate. Insertion of the i-gel was associated with one case of soft palate trauma which presented as minor bleed lasting for four hours. There was no significant difference between the PLMA, SLMA, i-gel, and LTS-D groups in the incidence of blood traces on the device. SLIPA was associated with significantly higher airway morbidity than PLMA, SLMA, and i-gel (*p* = 0.005).

## 4. Discussion

This study compared the performance of five different supraglottic airway devices with an additional mechanism for drainage/storage of gastric contents (2nd-generation SADs) in simulated field scenario when all devices were inserted by nonexperienced military personnel. The main findings are that the most suitable devices for use in this scenario are the Supreme LMA and i-gel airway and that the LTS-D and SLIPA have less favorable insertion parameters as well as other features. Important parameters for use in the field are the insertion success rate and speed of insertion. Both the SLMA and i-gel airway not only showed a high first-attempt insertion success rate and faster insertion times than other SADs but also were evaluated by the participants as most “easy to insert.” PLMA has a reasonable insertion profile but it possesses significant disadvantage compared to the other studied devices. The device is not available as a disposable version and the use of reusable device in the field is difficult. The i-gel may offer another potential advantage against the rest of these SADs. It can be used as a conduit for a tracheal tube placement such as with an intubating LMA [15].

The results of our study may be compared with other evidence related to 2nd-generation SADs. Ragazzi et al. compared insertion success rates and other parameters between the SLMA and i-gel when inserted by novice operators [14]. They found a significantly higher first-attempt insertion success rate in the SLMA group as well as a shorter insertion time and higher oropharyngeal seal pressures. They recommended the SLMA as the first-choice device in emergency situations. SLMA also showed faster insertion times than the classical LMA during a simulated CPR scenario [16]. Our results did not confirm this superiority of the SLMA over the i-gel, which is similar to the results of another study comparing these two devices in gynecological laparoscopies [17]. There is no data available comparing the LTS-D device with other supraglottic airways inserted by novices. One case series considered the LTS-D as a promising device for out-of-hospital emergency management when the operators were inexperienced in tracheal intubation [11]. These same authors reported a 96.8% total success rate when using this device in the prehospital setting, with a first-attempt insertion success rate of 78.3% [18]. LTS II showed significantly lower first-attempt success rate than the ProSeal LMA in anesthetized and paralyzed patients [19]. A manikin study compared performance and skill retention of airway management using various supraglottic airway devices versus tracheal intubation [20]. The novice operators showed better performance and retention of insertion skills at 3 months with the i-gel, laryngeal tube device (LT-D) than with the tracheal intubation. However, the results of manikin studies cannot be extrapolated to humans because they do not accurately reflect human anatomy. SLIPA showed similar insertion parameters when inserted by inexperienced operators to the 1st-generation SADs [21]. However, in another study, the SLIPA showed a significantly lower insertion success rate on the first attempt than those for the PLMA [22].

ERC guidelines [23] recommend that airway should be secured within 30 sec but this interval would be probably significantly longer in the field due to various factors such as single-handed lifesaver, difficult combat conditions, ban on the using of artificial light, or other unpredictable factors.

## 5. Limitations

This study has several limitations related mainly to its emphasis on military medicine. Simulated conditions differ significantly from real situations in the field. Field conditions are very stressful for military paramedics, unconscious victims may struggle, and there is often orofacial or neck trauma with bleeding present, causing airway management to be more difficult. The victims are also often found in various positions although they are subsequently moved to supine position for airway management procedures.

Furthermore other factors such as light conditions, neck immobilization, quality of training, or device insertion technique affect success rate significantly in out-of-hospital scenarios. Gastric tubes were also not inserted through the devices to drain gastric contents. We have decided against their insertion because one of the devices (SLIPA) does not allow gastric tube insertion and also because gastric tubes are not part of the emergency equipment carried by combat lifesavers. We also allowed participants to perform more than usual three attempts on the placement of device. Other limitations of the study include that postoperative airway morbidity and other patient complaints were not assessed. However, there has been no study to date published comparing these 2nd-generation supraglottic airway devices, with supposed lower incidence of gastric content aspiration in relation to airway management in the field, and the results of this study could become a starting point for further research projects evaluating the role of these devices in military medicine. Finally, few new devices, such as the 3gLM airway [24] or CPV Guardian Laryngeal Mask [25], have been invented in the last years and they may be used for similar comparative studies in the future.

## 6. Conclusions

The Supreme LMA and i-gel supraglottic airway seem to be the most convenient of the 2nd-generation supraglottic airway devices for insertion by relatively inexperienced military healthcare providers in a simulated low light scenario. Other devices tested showed either lower success rates of insertion or a significantly longer insertion time.

## Figures and Tables

**Table 1:** Main features of tested supraglottic airway devices.

Device	Sealing site	Sealing mechanism	Aspiration protection	Disposable version	Conduit for intubation	Pediatric sizes
ProSeal LMA	Perilaryngeal	Inflatable cuff	H/obstruction, drainage	No	No	Yes
Supreme LMA	Perilaryngeal	Inflatable cuff	H/obstruction, drainage	Yes	No	Yes
i-gel	Perilaryngeal	Wedged sealing	H/obstruction, drainage	Yes	Yes	Yes
SLIPA	Base of tongue	Wedged sealing	Storage, H/obstruction	Yes	No	No
LTS-D	Base of tongue	Inflatable cuff	D/obstruction, drainage	Yes	No	Yes

H/obstruction: high esophageal obstruction, D/obstruction: deep esophageal obstruction.

**(a):** 

Device	First-attempt success rate			
	Successful	Unsuccessful	Total	(%)
PLMA	85	16	101	84.2
SLMA	97	5	102	95.1
i-gel	87	13	100	87.0
SLIPA	66	34	100	66.0
LTS-D	79	23	102	77.5

**(b):** 

	PLMA versus SLMA	PLMA versus i-gel	PLMA versus SLIPA	PLMA versus LTS-D	SLMA versus i-gel	SLMA versus SLIPA	SLMA versus LTS-D	i-gel versus SLIPA	i-gel versus LTS-D	SLIPA versus LTS-D
*p*	0.012^*∗*^	0.689	0.003^*∗*^	0.285	0.081	0.0001^*∗*^	0.0004^*∗*^	0.0007^*∗*^	0.098	0.086

Differences marked with (*∗*) are statistically significant.

**Table 3:** Insertion times and oropharyngeal seal pressures (OSP).

Device	Insertion time (s, ±SD)	Oropharyngeal seal pressure (cm H_2_O, ±SD)
PLMA	109.6 (61.5)	29.2 (6.8)
SLMA	70.4 (32.5)	24.8 (6.1)
i-gel	74.4 (41.1)	25.3 (6.9)
SLIPA	98.5 (59)	23.7 (6.1)
LTS-D	107.3 (67.9)	29.5 (8.9)

Insertion time:

SLMA versus PLMA, SLMA versus SLIPA, and SLMA versus LTS-D: *p* = 0.001.

i-gel versus PLMA, i-gel versus LTS-D: *p* = 0.001, and i-gel versus SLIPA: *p* = 0.01.

Oropharyngeal seal pressures:

PLMA versus SLMA, PLMA versus i-gel, and PLMA versus SLIPA: *p* = 0.001.

LTS-D versus SLMA, LTS-D versus i-gel, and LTS-D versus SLIPA: *p* = 0.001.

**Table 4:** Coverage of the glottic opening (fibre optic assessment).

Device	Fibre optic assessment			
	1	2	3	4
PLMA	60 (64.5%)	14 (15.1%)	16 (17.2%)	3 (3.2%)
SLMA	53 (54.1%)	19 (19.4%)	22 (22.4%)	4 (4.1%)
i-gel	70 (72.2%)	16 (16.5%)	10 (10.3%)	1 (1%)
SLIPA	48 (58.5%)	9 (11%)	20 (24.4%)	5 (6.1%)
LTS-D	39 (41.1%)	20 (21.1%)	13 (13.7%)	23 (24.2%)

PLMA versus LTS-D: *p* = 0.001.

i-gel versus SLMA: *p* = 0.05, i-gel versus SLIPA: *p* = 0.05, and i-gel versus LTS-D: *p* = 0.001.

SLMA versus LTS-D: *p* = 0.001.

SLIPA versus LTS-D: *p* = 0.001.

**Table 5:** Ease of insertion (1: very easy, 2: easy, 3: neither easy nor difficult, 4: difficult, and 5: very difficult).

Device	Ease of insertion				
	1	2	3	4	5
PLMA	34.3%	44.4%	15.2%	5.1%	1%
SLMA	61.4%	30.7%	7.9%	0%	0%
i-gel	37.4%	48.5%	6.1%	6.1%	2%
SLIPA	7.1%	38.8%	24.5%	17.3%	12.2%
LTS-D	16.3%	43.9%	23.5%	13.3%	3.1%

SLMA versus PLMA, SLMA versus SLIPA, and SLMA versus LTS-D: *p* = 0.001, and SLMA versus i-gel: *p* = 0.01.

PLMA versus SLIPA: *p* = 0.001, PLMA versus LTS-D: *p* = 0.05.

i-gel versus SLIPA, i-gel versus LTS-D: *p* = 0.001, and SLIPA versus LTS-D: *p* = 0.05.

## Abbreviations

APL valve: Adjustable pressure limiting valve
BMI: Body mass index
CLS: Combat lifesaver
CPR: Cardiopulmonary resuscitation
ERC: European Resuscitation Council
etCO_2_: End-tidal carbon dioxide
GCS: Glasgow Coma Scale
LTS-D: Laryngeal Tube Suction-Disposable
OSP: Oropharyngeal seal pressure
PCV: Pressure controlled ventilation
PEEP: Positive end-expiratory pressure
PLMA: ProSeal laryngeal mask airway
SAD: Supraglottic airway device
SLIPA: Streamlined Liner of the Pharyngeal Airway
SLMA: Supreme laryngeal mask airway
TCCC: Tactical Combat Casualty Care.
